# Homonymous Hemianopia: A Rare Presentation of Secondary Central Nervous System Neurolymphomatosis

**DOI:** 10.7759/cureus.2708

**Published:** 2018-05-29

**Authors:** Fadil Awis Qarni, Evelyn Tai, Wan-Hazabbah WH, Azlan Husin

**Affiliations:** 1 Ophthalmology, Universiti Sains Malaysia, Kubang Kerian, MYS; 2 Department of Ophthalmology, Universiti Sains Malaysia, Kubang Kerian, MYS; 3 Medicine, Universiti Sains Malaysia, Kubang Kerian, MYS

**Keywords:** neurolymphomatosis, homonymous hemianopia, lymphoma, diffuse large b-cell lymphoma, non-hodgkin lymphoma

## Abstract

Neurolymphomatosis is an atypical complication of non-Hodgkin lymphoma and leukaemia involving infiltration of neurotropic neoplastic cells in the central or peripheral nervous system. A 28-year-old Malay lady with background diffuse large B-cell lymphoma stage IV presented with left homonymous hemianopia associated with cognitive function deterioration. Her best corrected visual acuity was 6/9 in both eyes. Magnetic resonance imaging (MRI) of the brain showed a lesion suggestive of secondary lymphomatous infiltration of the splenium of corpus callosum. The patient underwent chemotherapy, after which repeated MRI showed a reduction in the lesion size. Homonymous hemianopia is a rare presentation of secondary central nervous system neurolymphomatosis. A comprehensive history, physical examination, and radiological imaging are essential to establish the diagnosis in patients presenting with visual field defects.

## Introduction

Neurolymphomatosis is an atypical complication of non-Hodgkin lymphoma and leukaemia characterized by infiltration of neurotropic neoplastic cells in the central or peripheral nervous system [1-2]. It may be primary or secondary to metastases [3-4], and usually manifests as axonal polyneuropathy corresponding to the site of peripheral nervous system invasion [5]. We report a rare case of secondary neurolymphomatosis presenting with homonymous hemianopia.

## Case presentation

A 28-year-old lady was referred to the neuro-ophthalmology clinic of Hospital Universiti Sains Malaysia for progressive bilateral visual disturbances over a period of three months. The patient was diagnosed to have diffuse large B-cell lymphoma stage IV with metastases to the breast, pancreas, lungs, and ovaries. The diagnosis was made a year before when she presented with right breast lumps, which were subsequently biopsied.

She completed six cycles of R-CHOP (rituximab, cyclophosphamide, doxorubicin, vincristine, and prednisone) together with intrathecal methotrexate, dexamethasone, and cytosine arabinoside over a period of five months after the diagnosis. Four months later, she developed right upper limb neuropathic pain and was diagnosed with peripheral neurolymphomatosis of the right brachial plexus. Positron emission tomography with fluoro-D-glucose integrated with computed tomography (FDG-PET/CT) showed no central or peripheral nervous system involvement. She was initiated on methotrexate, but defaulted after the eighth cycle.

Three months later, she developed gradual onset of visual disturbances in both eyes, described as a loss of the left visual field in both eyes. She denied seeing any floaters or flashes of light. The symptoms worsened a month prior to presentation, associated with short-term memory loss and emotional lability. There was no history of fits, body weakness, or sensory deficits. There were also no symptoms of high intracranial pressure such as headache, nausea, or vomiting. She had no diplopia, dyschromatopsia, or metamorphopsia. She denied any recent trauma.

**Figure 1:**
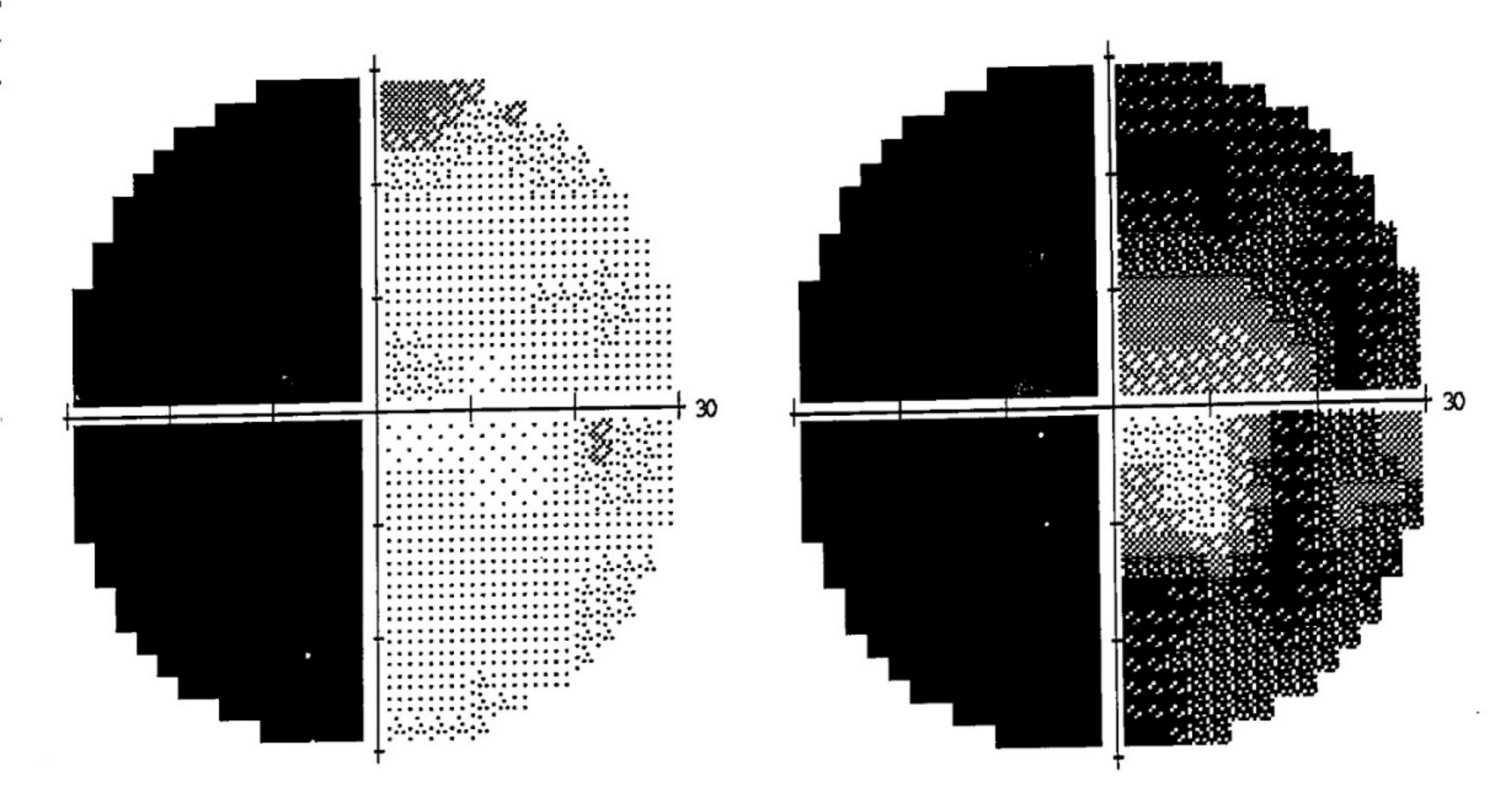
30-2 Humphrey visual field perimetry Complete left homonymous hemianopia.

On examination, best-corrected visual acuity was 6/9 in both eyes. Confrontation visual field testing showed left homonymous hemianopia. Ishihara color vision testing was normal. There was no afferent pupillary defect. Both anterior and posterior segments were unremarkable. Humphrey automated visual field confirmed a dense left homonymous hemianopia (Figure 1).

**Figure 2:**
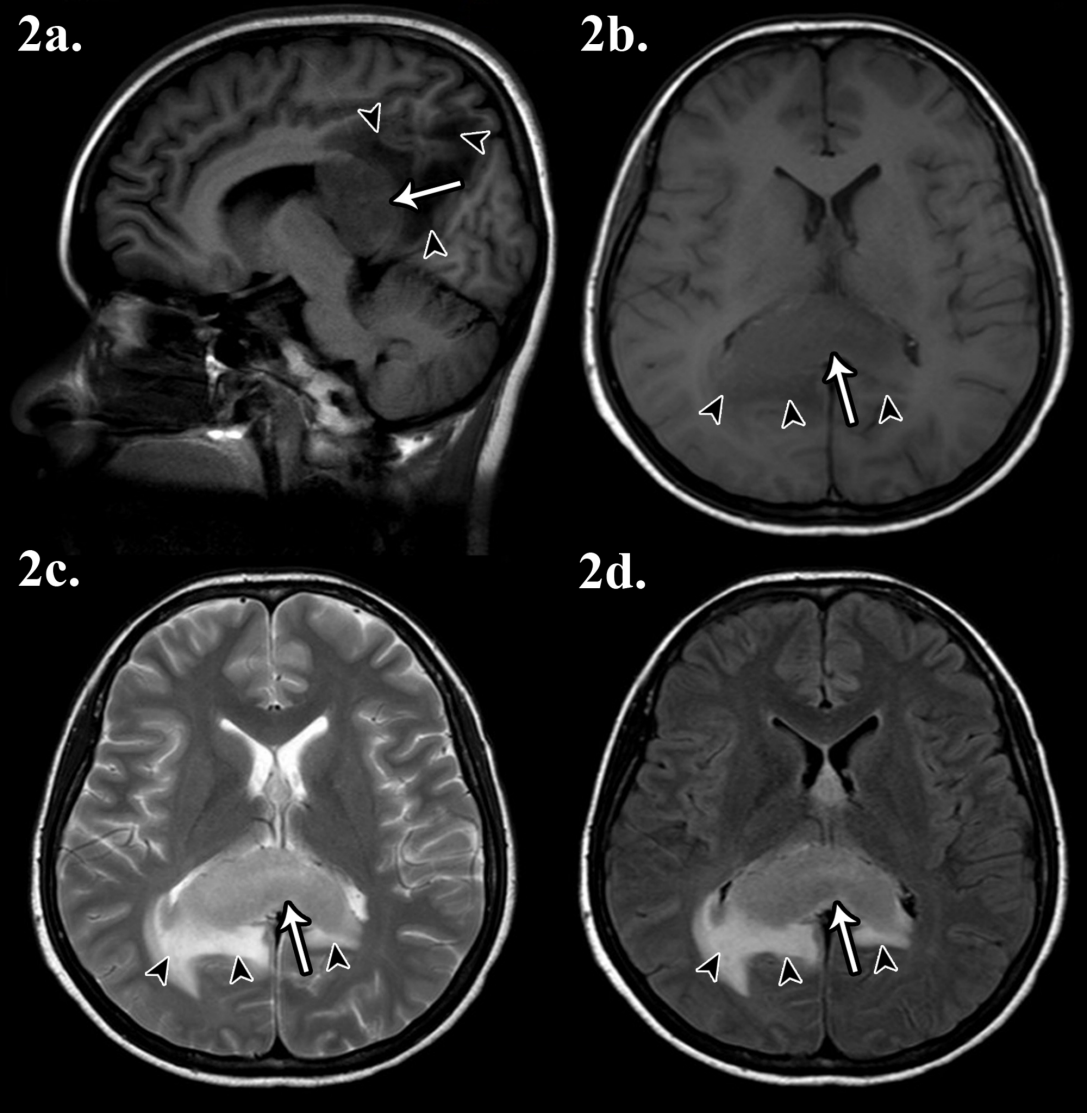
Magnetic resonance imaging (MRI) of the brain 2a) T1-weighted sagittal imaging; 2b) T1-weighted axial imaging; 2c) T2-weighted axial imaging; 2d) fluid attenuation inversion recovery (FLAIR) sequence imaging. A well-defined mass is visible in the splenium of corpus callosum (white arrow), crossing the midline. The mass is isointense on T1, hyperintense on T2, and hyperintense on FLAIR. There is associated white matter oedema (black arrowheads).

Magnetic resonance imaging (MRI) of the brain revealed a well-defined mass in the splenium of corpus callosum associated with white matter oedema, suggestive of metastases (Figure 2).

**Figure 3:**
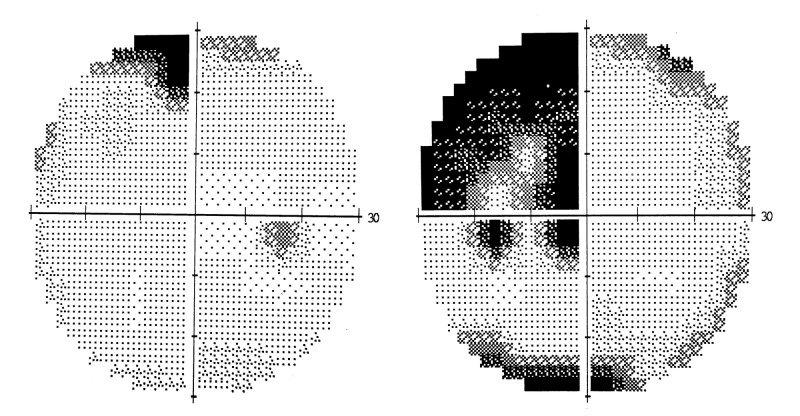
30-2 Humphrey visual field perimetry post chemotherapy Significant improvement in the previously noted left homonymous hemianopia.

She was diagnosed with secondary central nervous system neurolymphomatosis and started on a regime of rituximab, ifosfamide, carboplatin, and etoposide. She was also given another cycle of intrathecal methotrexate, steroid (dexamethasone), and cytarabine. After completion of three cycles of chemotherapy over a period of two months, her visual field improved significantly (Figure 3).

**Figure 4:**
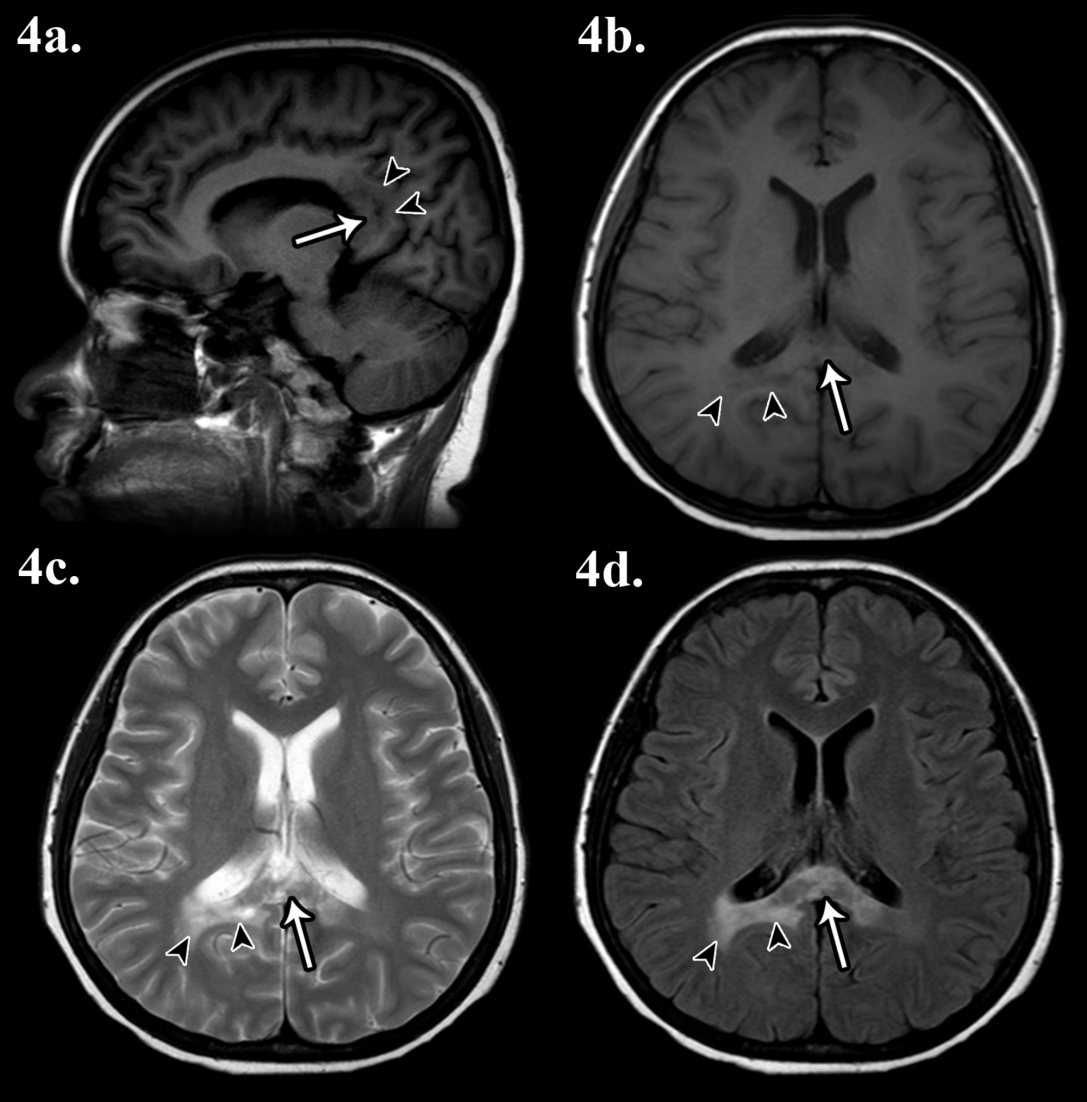
Magnetic resonance imaging (MRI) of the brain post chemotherapy 4a) T1-weighted sagittal imaging; 4b) T1-weighted axial imaging; 4c) T2-weighted axial imaging; 4d) fluid attenuation inversion recovery (FLAIR) sequence imaging. Marked reduction in the size of the previously seen mass at the splenium of corpus callosum (white arrow), ​​​​​​with reduced white matter oedema (black arrowheads).

MRI after completion of chemotherapy showed that the mass in the splenium of corpus callosum had reduced markedly in size (Figure 4).

## Discussion

Neurolymphomatosis refers to the infiltration of malignant lymphocytes into the central or peripheral nervous system [2,6]. Although its aetiology is unclear, it has been observed as a rare extranodal manifestation of non-Hodgkin lymphoma [7-8]. It has also been reported in leukaemic patients [9].

The presentation of neurolymphomatosis depends on the location of malignant lymphocyte infiltration. In cases of central nervous system involvement, patients tend to present with visual complaints. Zhu et al. reported an isolated lymphoma of the optic nerve, chiasm and tract in a patient who presented with progressive visual impairment and eventually became blind in both eyes [3]. Another patient with primary cerebral non-Hodgkin lymphoma presented with two months history of dizziness and progressive left hemiparesis, associated with right eye blurred vision and floaters [10]. Bitemporal hemianopia has been reported in a neurolymphomatosis patient with multiple intracranial hyperdense lesions and a large mass occupying the suprasellar region and the third ventricle [10]. Homonymous hemianopia is an uncommon presentation of this condition, and is more commonly associated with stroke and trauma [11].

MRI is a widely used diagnostic modality for neurolymphomatosis. However, FDG-PET/CT is more sensitive for the diagnosis than MRI [1-2,6]. A retrospective study involving 50 neurolymphomatosis patients showed that FDG-PET/CT was positive in 84% of cases, among whom MRI only detected 74% of cases [6]. Other diagnostic modalities include nerve biopsy and cerebrospinal fluid cytology. The latter has low sensitivity (40%), while nerve biopsy is highly sensitive (88%) but runs the risk of associated morbidity [6].

The prognosis of neurolymphomatosis correlates with its response to treatment. Grisariu et al. showed that with treatment, approximately 46% of patients achieve clinical improvement [6]. Prompt and aggressive chemotherapy (systemic and intrathecal) with or without radiotherapy is the mainstay of treatment [1].

## Conclusions

Homonymous hemianopia is a rare presentation of secondary central nervous system neurolymphomatosis. Patients with underlying lymphoma who present with visual field defects should be investigated for central nervous system metastases. A comprehensive history, physical examination, and radiological imaging are essential in all such patients.
